# BioThings Explorer: a query engine for a federated knowledge graph of biomedical APIs

**DOI:** 10.1093/bioinformatics/btad570

**Published:** 2023-09-14

**Authors:** Jackson Callaghan, Colleen H Xu, Jiwen Xin, Marco Alvarado Cano, Anders Riutta, Eric Zhou, Rohan Juneja, Yao Yao, Madhumita Narayan, Kristina Hanspers, Ayushi Agrawal, Alexander R Pico, Chunlei Wu, Andrew I Su

**Affiliations:** Department of Integrative Structural and Computational Biology, The Scripps Research Institute, La Jolla, CA 92037, United States; Department of Integrative Structural and Computational Biology, The Scripps Research Institute, La Jolla, CA 92037, United States; Department of Integrative Structural and Computational Biology, The Scripps Research Institute, La Jolla, CA 92037, United States; Department of Integrative Structural and Computational Biology, The Scripps Research Institute, La Jolla, CA 92037, United States; Data Science and Biotechnology, Gladstone Institutes, University of California, San Francisco, CA 94158, United States; Department of Integrative Structural and Computational Biology, The Scripps Research Institute, La Jolla, CA 92037, United States; Department of Integrative Structural and Computational Biology, The Scripps Research Institute, La Jolla, CA 92037, United States; Department of Integrative Structural and Computational Biology, The Scripps Research Institute, La Jolla, CA 92037, United States; Department of Integrative Structural and Computational Biology, The Scripps Research Institute, La Jolla, CA 92037, United States; Data Science and Biotechnology, Gladstone Institutes, University of California, San Francisco, CA 94158, United States; Data Science and Biotechnology, Gladstone Institutes, University of California, San Francisco, CA 94158, United States; Data Science and Biotechnology, Gladstone Institutes, University of California, San Francisco, CA 94158, United States; Department of Integrative Structural and Computational Biology, The Scripps Research Institute, La Jolla, CA 92037, United States; Department of Integrative Structural and Computational Biology, The Scripps Research Institute, La Jolla, CA 92037, United States

## Abstract

**Summary:**

Knowledge graphs are an increasingly common data structure for representing biomedical information. These knowledge graphs can easily represent heterogeneous types of information, and many algorithms and tools exist for querying and analyzing graphs. Biomedical knowledge graphs have been used in a variety of applications, including drug repurposing, identification of drug targets, prediction of drug side effects, and clinical decision support. Typically, knowledge graphs are constructed by centralization and integration of data from multiple disparate sources. Here, we describe BioThings Explorer, an application that can query a virtual, federated knowledge graph derived from the aggregated information in a network of biomedical web services. BioThings Explorer leverages semantically precise annotations of the inputs and outputs for each resource, and automates the chaining of web service calls to execute multi-step graph queries. Because there is no large, centralized knowledge graph to maintain, BioThings Explorer is distributed as a lightweight application that dynamically retrieves information at query time.

**Availability and implementation:**

More information can be found at https://explorer.biothings.io and code is available at https://github.com/biothings/biothings_explorer.

## 1 Introduction

While downloadable files are the most common way to share biomedical data, application programming interfaces (APIs) are another popular and powerful mechanism for data dissemination. Accessing data through APIs has many complementary advantages relative to downloading local copies of data. APIs typically allow users to query for specific subsets of the data that are of interest. API queries are often highly indexed, leading to efficient data retrieval. Finally, API access allows for easy incorporation of the most up-to-date data into other computational applications or workflows.

While APIs offer many advantages in terms of data accessibility, these advantages do not immediately translate into efficient data integration. APIs generally follow some common architectures and protocols [e.g. representational state transfer (REST), output in JavaScript Object Notation (JSON)], but alignment at this technical level does not guarantee either syntactic or semantic interoperability. For example, APIs can use different identifiers for the same gene, different data structures to represent gene attributes, and different terms to describe the relationships between biomedical entities.

There have been some efforts to define and enforce semantic and syntactic standards to achieve data interoperability. Examples of this approach include the Beacon API from the GA4GH consortium (Rambla *et al.* 2022) and the DAS specification for sharing annotations of genomic features (Dowell *et al.* 2001). These efforts rely on the active participation of API developers in adopting a community standard for their API endpoints.

Here, we explore the use of semantically precise API annotations as a complementary approach to achieving API interoperability. We divided this work into two components. First, we created an extension of the widely used OpenAPI annotation standard (http://spec.openapis.org/oas/v3.1.0) to semantically describe the APIs’ inputs and outputs, and a registry to organize these API annotations. Second, we created an application called BioThings Explorer to consume the API metadata and to execute multi-hop graph queries that span multiple APIs. Together, this architecture allows users to query a large, federated knowledge graph based on an interconnected network of biomedical APIs. This federated design offers a unique approach for creating knowledge graphs that is complementary to the more common strategy of centralization and local data integration.

## 2 A registry of semantically annotated APIs

The first step in creating a network of interoperable APIs is to annotate each API in a semantically precise way. We built this API annotation system on the OpenAPI specification, the *de facto* standard for documenting API metadata in a human- and machine-readable format. OpenAPI describes basic API metadata (e.g. title, description, version, contact info), as well as key information on the operation of the API endpoints (e.g. server URL, endpoint input parameters, endpoint response schemas).

However, this basic OpenAPI specification does not include key domain-specific information that is necessary to facilitate downstream API interoperability. Therefore, we defined an OpenAPI extension to capture semantically precise annotations of each API endpoint. These annotations include the semantic types and identifier namespaces of biomedical entities that are both used in querying (inputs) and found in the response (outputs), the JSON path to the output identifier values in the JSON response, and the predicate describing the relationship between the input and output entities (Supplementary Fig. S1).

We also chose a strategy to map arbitrary JSON data structures to an established biological data model. In this effort, we mapped API output to the Biolink Model (Unni *et al.* 2022), a community-developed data model that was adopted and extended by the NCATS Translator consortium (Fecho *et al.* 2022). The Biolink model defines the universe of semantic types that can be used in BioThings Explorer, the allowed identifier systems for each semantic type and the allowed predicates. We provided a guide to writing these API annotations in our GitHub repository (https://github.com/biothings/biothings_explorer/blob/main/docs/README-writing-x-bte.md).

**Figure 1.**
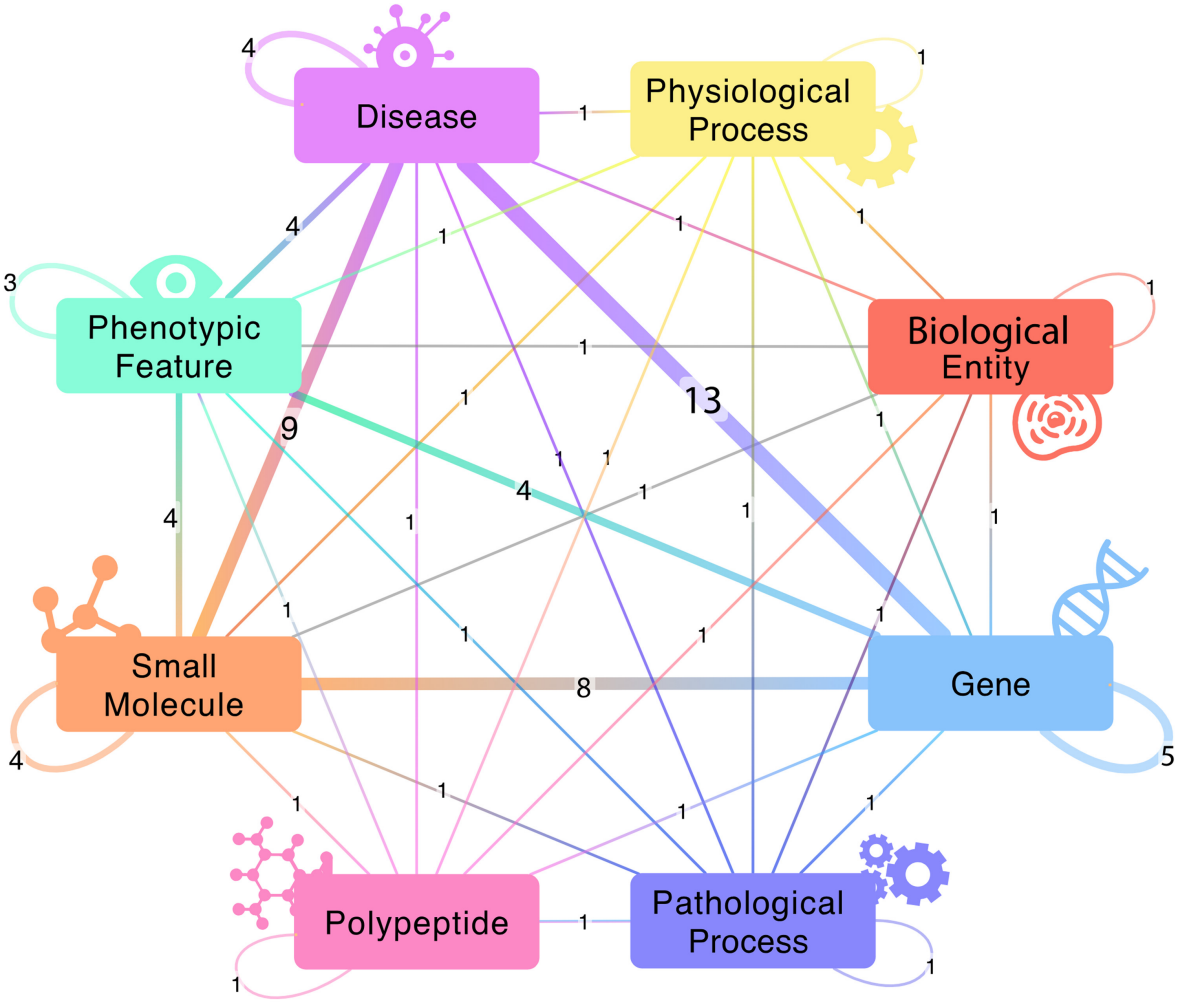
A visualization of the meta-KG for BioThings Explorer. The nodes in this graph are the semantic types of biomedical entities that BioThings Explorer can retrieve associations between (limited to the top eight most common semantic types). The edges between nodes show what associations between biomedical entities exist in the semantic API network that is accessible through BioThings Explorer. The edge label shows the number of APIs that can retrieve those types of associations, which is also represented by the edge width.

To annotate and catalog APIs with our OpenAPI extension, we leveraged the SmartAPI registry (https://smart-api.info/) (Zaveri *et al.* 2017). We created SmartAPI registrations for 34 APIs with semantic annotations. The selection of these APIs that are queried by BioThings Explorer is set in a local, instance-specific configuration file. This collection of API annotations can be thought of as a “meta-knowledge graph” (meta-KG), where the nodes represent types of biomedical entities (genes, diseases, drugs) and the edges represent APIs that describe relationships between two types of biomedical entities. The SmartAPI meta-KG currently contains 35 nodes and 1817 edges. (The complete meta-KG is shown in Supplementary Table S1 and a partial rendering is shown in Fig. 1.)

## 3 API interoperability using BioThings Explorer

**Figure 2.**
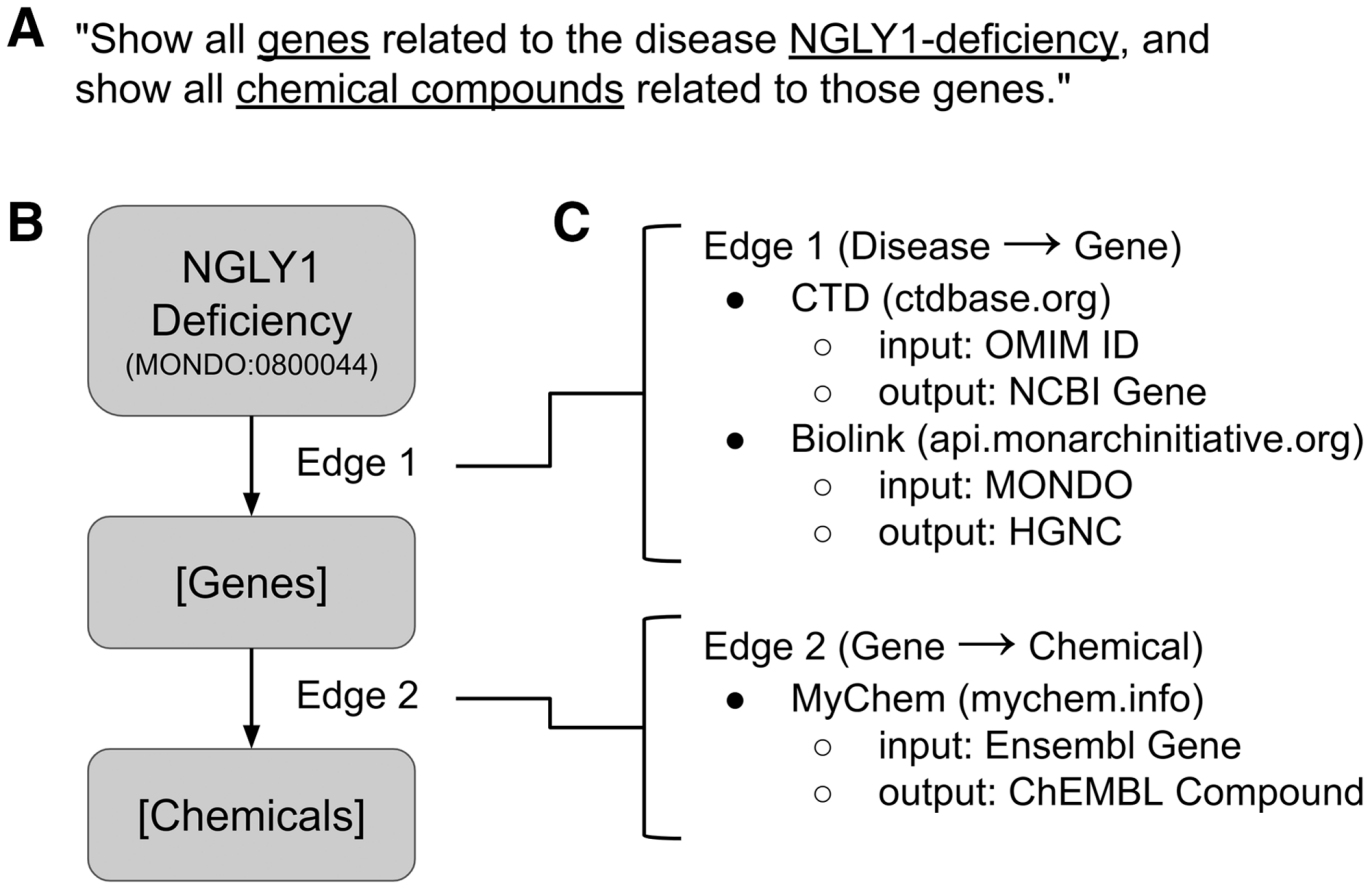
Deconstruction of a query in BioThings Explorer. (A) A free-text representation of a query that can be answered by BioThings Explorer. (B) The graph representation of the same query. The exact syntax of this graph query is specified in the Translator Reasoner API standard described in Fecho *et al.* (2022) and shown in Supplementary Fig. S2. (C) The deconstruction of the graph query into multiple API calls by consulting the meta-KG in the SmartAPI registry.

The second step in creating our federated biomedical knowledge graph was to create BioThings Explorer, an engine to autonomously query the SmartAPI meta-KG, query the annotated APIs to retrieve associations between biomedical entities, and integrate those APIs’ responses. The input to BioThings Explorer is a query graph and the syntax for encoding the query graph was defined by the NCATS Translator consortium (Fecho *et al.* 2022). The topology of the query graph and the constraints on its nodes and edges define the query (Fig. 2).

BioThings Explorer executes the query in three distinct phases: query-path planning, query-path execution, and integration and scoring.

### 3.1 Query-path planning

For every edge in a query graph, BioThings Explorer consults the SmartAPI registry for APIs that serve those types of associations (Fig. 2). For example, in Fig. 1, associations between diseases and genes can be found using APIs from the Comparative Toxicogenomics Database (Davis *et al.* 2023) and the Biolink API from the Monarch Initiative (Mungall *et al.* 2017, Unni *et al.* 2022), while associations between genes and chemicals can be found using MyChem.Info (https://mychem.info/) (Lelong *et al.* 2022). The sequence of API calls that can satisfy the original query is a “query-path plan.”

### 3.2 Query-path execution

In this phase, BioThings Explorer programmatically and autonomously executes each query in each query-path plan based on the semantic annotations for each API identified in the previous phase. BioThings Explorer calls each API, using the SmartAPI annotation to construct calls with the correct syntax and appropriate input identifier, and maps the API responses to the Biolink Model (Unni *et al.* 2022). BioThings Explorer also performs ID-to-object translation, which facilitates the chaining of API calls from one step in the query-path to the next step. This ID translation step is critical when successive APIs in the query-path plan use different identifiers to represent the same biomedical entity (e.g. NCBI Gene ID versus Ensembl Gene ID). ID translation is currently handled by the Translator Node Normalizer (https://github.com/NCATSTranslator/Translator-All/wiki/Node-Normalizer). The output of this phase is a set of edges for each step of the query-path, which represent the associations between biomedical entities retrieved from the APIs.

### 3.3 Integration and scoring

In this final phase, these sets of edges from the API queries are assembled into result sub-graphs, each of which matches the topology of the query graph. Each result is then scored based on a variety of factors including number of paths, length of paths, and semantic similarity between concepts based on the Normalized Google Distance (Cilibrasi and Vitanyi 2007).

## 4 Deployment and usage

The BioThings Explorer knowledge graph is entirely composed from a federated network of APIs. Because there is no local assembly and storage of a large knowledge graph, BioThings Explorer is a very lightweight application that can be easily deployed on almost any standard personal computer. The ability of every user to create a local instance of BioThings Explorer removes centralized bottlenecks associated with large queries and/or heavy usage. The code repository that describes the installation process is at https://github.com/biothings/biothings_explorer. BioThings Explorer currently relies on two external dependencies—the Node Normalizer service and the Biolink Model (Unni *et al.* 2022). However, nothing in the BioThings Explorer architecture is reliant on these specific tools, so these dependencies can be substituted for alternatives if desired.

For users who prefer not to create a local instance of BioThings Explorer, we also maintain a community instance for general use through the NCATS Translator Consortium (https://explorer.biothings.io/).

## 5 Discussion

Integration of existing data from multiple disparate sources is a key step in assessing the state of current knowledge. There are many existing efforts to create biomedical knowledge graphs by integrating locally downloaded data and standardizing it using a common data model (Himmelstein *et al.* 2017; Fecho *et al.* 2021; Mayers *et al.* 2022; Wood *et al.* 2022; Morris *et al.* 2023). These efforts result in centralized knowledge graphs of substantial size, often with millions of nodes and tens of millions of edges.

BioThings Explorer offers a unique strategy for data integration, focusing on creating a federated knowledge graph by semantically annotating APIs. Rather than bringing all data into a massive, centralized graph database, this federated design instead allows knowledge to remain behind each resource’s API. Data are retrieved at query time by dynamically executing API calls and semantically parsing the results. This architecture functionally separates data dissemination (through API creation) from data modeling and data integration (through semantic annotations).

This approach has several advantages. First, by moving the requirements for interoperability from *implementation in code* to *semantic API annotation*, we significantly lower the barrier to participation in our API ecosystem. Second, by separating these roles into distinct layers, we promote the overall modularity of our system. These components can develop and evolve in parallel, and these two roles can even be undertaken by separate teams (e.g. one team semantically annotates an API that was created by another team). Third, this design facilitates an iterative approach to API annotation. Developers and API annotators can first provide a minimal set of API metadata, which can later be extended based on future needs and use cases.

The federated design of BioThings Explorer also has some notable limitations. First, our OpenAPI extensions in SmartAPI to semantically annotate APIs only work on APIs that follow the REST protocol and provide output in JSON format. Second, because the entire federated KG is never instantiated in a single place, reasoning and scoring methods that rely on having the entire knowledge graph in memory cannot be used with BioThings Explorer.

In sum, we believe that knowledge graphs enable many exciting use cases in biomedical research (Nicholson and Greene 2020), and that BioThings Explorer is complementary to existing approaches for assembling knowledge graphs, offering powerful and unique capabilities for both scientific data analysts and tool developers.

## Supplementary Material

btad570_Supplementary_Data

## Data Availability

BioThings Explorer is implemented as a NodeJS application. The primary repository for the BioThings Explorer project is at https://github.com/biothings/biothings_explorer, which in turn links to and incorporates other repositories as sub-modules. All code is released under the Apache 2.0 open-source software license.

## References

[btad570-B1] Cilibrasi RL , Vitanyi PMB. The google similarity distance. IEEE Trans Knowl Data Eng 2007;19:370–83.

[btad570-B2] Davis AP , Wiegers TC, Johnson RJ et al Comparative toxicogenomics database (CTD): update 2023. Nucleic Acids Res 2023;51:D1257–62. 36169237 10.1093/nar/gkac833 PMC9825590

[btad570-B3] Dowell RD , Jokerst RM, Day A et al The distributed annotation system. BMC Bioinformatics 2001;2:7. 11667947 10.1186/1471-2105-2-7 PMC58584

[btad570-B4] Fecho K , Bizon C, Miller F et al A biomedical knowledge graph system to propose mechanistic hypotheses for real-world environmental health observations: cohort study and informatics application. JMIR Med Inform 2021;9:e26714. 34283031 10.2196/26714 PMC8335603

[btad570-B5] Fecho K , Thessen AE, Baranzini SE et al Progress toward a universal biomedical data translator. Clin Transl Sci 2022;15:1838–47. 35611543 10.1111/cts.13301 PMC9372428

[btad570-B6] Himmelstein DS , Lizee A, Hessler C et al Systematic integration of biomedical knowledge prioritizes drugs for repurposing. Elife 2017;6:e26726. 10.7554/eLife.26726 PMC5640425 28936969

[btad570-B7] Lelong S , Zhou X, Afrasiabi C et al BioThings SDK: a toolkit for building high-performance data APIs in biomedical research. Bioinformatics 2022;38:2077–9. 35020801 10.1093/bioinformatics/btac017 PMC8963279

[btad570-B8] Mayers M , Tu R, Steinecke D et al Design and application of a knowledge network for automatic prioritization of drug mechanisms. Bioinformatics 2022;38:2880–91. 35561182 10.1093/bioinformatics/btac205 PMC9113361

[btad570-B9] Morris JH , Soman K, Akbas RE et al The scalable precision medicine open knowledge engine (SPOKE): a massive knowledge graph of biomedical information. Bioinformatics 2023;39(2):btad080. 10.1093/bioinformatics/btad080 PMC9940622 36759942

[btad570-B10] Mungall CJ , McMurry JA, Köhler S et al The monarch initiative: an integrative data and analytic platform connecting phenotypes to genotypes across species. Nucleic Acids Res 2017;45:D712–22. 27899636 10.1093/nar/gkw1128 PMC5210586

[btad570-B11] Nicholson DN , Greene CS. Constructing knowledge graphs and their biomedical applications. Comput Struct Biotechnol J 2020;18:1414–28. 32637040 10.1016/j.csbj.2020.05.017 PMC7327409

[btad570-B12] Rambla J , Baudis M, Ariosa R et al Beacon v2 and Beacon networks: a ‘lingua franca’ for federated data discovery in biomedical genomics, and beyond. Hum Mutat 2022;43:791–9. 35297548 10.1002/humu.24369 PMC9322265

[btad570-B13] Unni DR , Moxon SAT, Bada M et al Biolink Model: a universal schema for knowledge graphs in clinical, biomedical, and translational science. Clin Transl Sci 2022;15:1848–55. 36125173 10.1111/cts.13302 PMC9372416

[btad570-B14] Wood EC , Glen AK, Kvarfordt LG et al RTX-KG2: a system for building a semantically standardized knowledge graph for translational biomedicine. BMC Bioinformatics 2022;23:400. 36175836 10.1186/s12859-022-04932-3 PMC9520835

[btad570-B15] Zaveri A , Dastgheib S, Wu C et al smartAPI: towards a more intelligent network of web APIs. In: Blomqvist, E., Maynard, D., Gangemi, A., et al. (eds) The Semantic Web. ESWC 2017. Lecture Notes in Computer Science, 2017, 154–69. https://link.springer.com/chapter/10.1007/978-3-319-58451-5_11#chapter-info.

