# Supplementary material for: BioThings Explorer: a query engine for a federated knowledge graph of biomedical APIs
Source: Bioinformatics. 2023 Sep 14;39(9):btad570. doi: 10.1093/bioinformatics/btad570 (PMC11015316; doi:10.1093/bioinformatics/btad570)
Supplement: btad570_Supplementary_Data [file btad570_supplementary_data.zip › supp_fig_1.pdf]

A)

https://www.ncbi.nlm.nih.gov/research/bionlp/litvar/api/v1/entity/litvar/rs121913527%23%23?format=json

B)


```
{
    "_id": "litvar@rs121913527##",
    "id": "rs121913527##",
    "db": "litvar",
  ▶ "years": [ … ], // 15 items
  ▶ "diseases": { … }, // 5 items
    "concept": "variant",
  ▶ "data": { … }, // 8 items
    "hgvs": "c.146A>T",
    "rsid": "rs121913527",
  ▶ "links": [ … ], // 1 item
  ▶ "all_hgvs": [ … ], // 18 items
    "hgvs_prot": "p.A146T",
    "weight": 1.996996996996997,
    "pmids_count": 333,
    "first_published_year": 2006,
    "name": "c.146A>T",
  ▼ "gene": {
        "id": 3845,
        "name": "KRAS"
    }
}
```


C)


```
x-bte-kgs-operations:
  variant_located_in_gene:
  - supportBatch: false
    useTemplating: true
    inputs:
    - id: DBSNP
      semantic: SequenceVariant
    parameters:
      variantid: "{{ queryInputs | rmPrefix() }}%23%23"
    outputs:
    - id: NCBIGene
      semantic: Gene
    predicate: is_sequence_variant_of
    source: "infores:dbsnp"
    response_mapping:
      "$ref": "#/components/x-bte-response-mapping/variant_located_in_gene"
x-bte-response-mapping:
  variant_located_in_gene:
    NCBIGene: gene.id
    ref_url: links.url
```


Supplemental Figure 1. Biothings Explorer uses extensions to the OpenAPI specification to semantically annotate APIs. These annotations include the semantic types and identifier namespaces of the biomedical entities that are used in querying (inputs) and found in the response (outputs), the JSON path to the output identifiers in the JSON response, and the predicate describing the relationship between the input and output entities. The allowed semantic types and the predicates are defined by the Biolink Model. This figure illustrates these extensions for an API serving content for LitVar. A) The LitVar API can be called using this syntax; the annotation specifies that DBSNP IDs (highlighted in yellow) should be used as the input in queries to this API. B) The API returns a JSON object with a gene related to the variant specified in the query (highlighted in green). C) The `x-kgs-operations` section of the SmartAPI annotation specifies that the data retrieved by the LitVar API relates "SequenceVariants" (identified using DBSNP IDs) to "Genes" (identified using NCBIGene IDs). The `x-bte-response-mapping` portion of the SmartAPI annotation specifies that the NCBIGene IDs of the related genes can be found in the data under the JSON path "gene.id".
