# Supplementary material for: BioThings Explorer: a query engine for a federated knowledge graph of biomedical APIs
Source: Bioinformatics. 2023 Sep 14;39(9):btad570. doi: 10.1093/bioinformatics/btad570 (PMC11015316; doi:10.1093/bioinformatics/btad570)
Supplement: btad570_Supplementary_Data [file btad570_supplementary_data.zip › supp_fig_2.pdf]

```json
{
    "message": {
        "query_graph": {
            "nodes": {
                "node0": {
                    "categories": ["biolink:Disease"],
                    "ids": ["MONDO:0800044"]
                },
                "node1": {
                    "categories": ["biolink:Gene"]
                },
                "node2": {
                    "categories": ["biolink:ChemicalEntity"]
                }
            },
            "edges": {
                "edge1": {
                    "subject": "node0",
                    "object": "node1"
                },
                "edge2": {
                    "subject": "node1",
                    "object": "node2"
                }
            }
        }
    }
}
```

Supplemental Figure 2. Here, we depict the same query that is described in **Figure 1**, but using the Translator Reasoner API (TRAPI) standard. It is this TRAPI query that is actually used as input to BioThings Explorer. Note that the allowed semantic types (under the "categories" key) are defined by the Biolink Model.
